# A Comprehensive Analysis of the Use of SFRC in Structures and Its Current State of Development in the Construction Industry

**DOI:** 10.3390/ma15197012

**Published:** 2022-10-10

**Authors:** Dhanasingh Sivalinga Vijayan, Arvindan Sivasuriyan, Devarajan Parthiban, Aleksandra Jakimiuk, Hydayatullah Bayat, Anna Podlasek, Magdalena Daria Vaverková, Eugeniusz Koda

**Affiliations:** 1 Civil Engineering, Aarupadai Veedu Institute of Technology, Vinayaka Missions Research Foundation, Chennai 603104, India; 2 Institute of Civil Engineering, Warsaw University of Life Sciences—SGGW, 02-787 Warsaw, Poland; 3 Faculty of AgriSciences, Mendel University in Brno, 613 00 Brno, Czech Republic

**Keywords:** steel fiber, concrete, structural members, blast loading, durability

## Abstract

In recent years, concrete technology has advanced, prompting engineers and researchers to adopt advanced materials to improve strength and durability. Steel-fiber-reinforced concrete (SFRC) represents the substantial modification of concrete materials to improve their structural properties, particularly their flexural and tensile strength. Whether SFRC is stronger than conventional concrete depends on a variety of variables, including the volume, size, percentage, shape, and distribution of fibers. This article provides a comprehensive discussion of the properties of SFRC, such as durability, fire resistance, and impact resistance or blast loading, as well as the application of SFRC in structural members including beams, columns, slabs, and walls. The application of steel fibers in various types of concrete, including pre-stressed, pre-cast, self-compacting, and geopolymer concrete, was also examined in this comparative analysis review, and recommendations for the future scope of SFRC were identified.

## 1. Introduction

**Figure 1:**
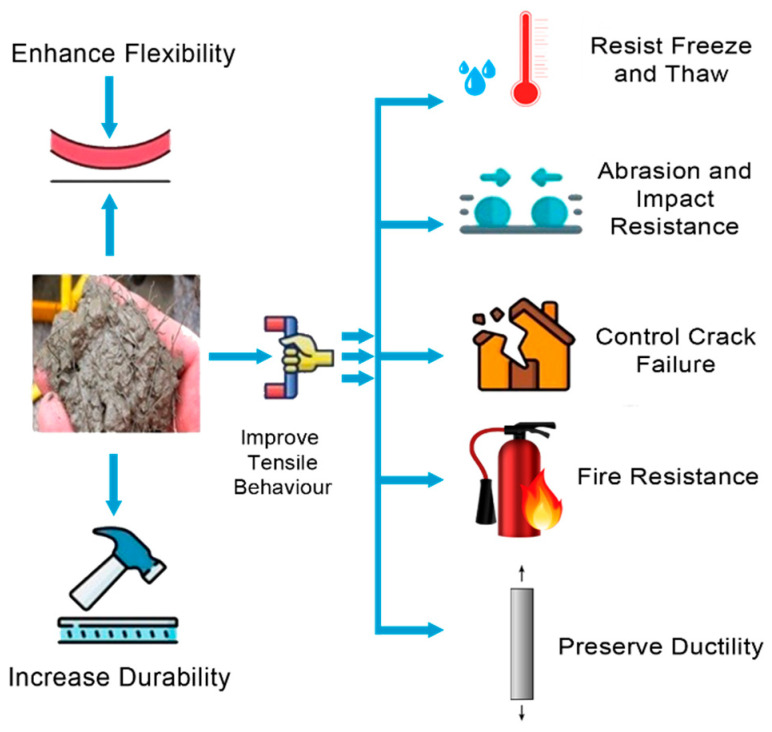
The efficiency of steel fibers in concrete.

Advancement in material science has become vital in the recent years. Concrete is one of the most significant materials globally, being an effective material for several applications, such as building dams, bridges, buildings, tunnels, and many other structures. Concrete, despite being considered the most common building material above other materials such as steel, wood, or composite systems, has its disadvantages, including its low tensile strength and brittleness [1,2]. Improving concrete technology by incorporating steel fibers can achieve concrete materials with extreme strength and durability. Steel fibers are a versatile material available in different sizes, shapes, and volumes; hence, they should be properly distributed inside the concrete to reach optimum flexural and tensile strength. Cement composites are fragile, making them susceptible to cracking at very low levels of tension. As reinforcements, steel bars, fibers, and meshes have been widely employed to address this problem. For several decades, a great deal of research has examined the use of various fibers in cement-based composites [3,4,5,6,7,8]. Numerous efforts have been made so far [9,10,11] to determine the efficacy of fibers in terms of their post-crack energy absorption capacity and crack arrest capability in reinforced concrete. Steel-fiber-reinforced concrete (SFRC) is one of the most auspicious new structural materials, incorporating randomly dispersed steel fibers of diverse geometries and sizes into the cement matrix to solve the challenges of the brittleness and crack susceptibility of cementitious composites [12]. Each year, approximately 0.3 million tonnes of steel fibers are brought to market on a global scale, and it is anticipated that this number will rise by 20% annually [13]. In addition, about 90% of these industrial steel fibers are accessible in a variety of forms, geometries, and surface textures [10]. While most of these steel fiber geometrical forms have been abandoned in recent years, some of them have proved useful for manufacturing concrete [14,15]. A vast number of steel businesses are presently functioning on a global scale, and each of them not only consumes substantial amounts of fossil fuels and energy, but also plays a significant role in the emission of greenhouse gases [16]. Due to the negative environmental impacts of the consumption of natural resources and the emission of CO_2_ during the industrial manufacture of steel fibers, major research efforts have been undertaken to replace these expensive steel fibers with sustainable and cost-effective alternatives. Over the past decade, recycled steel fibers (RSFs) have been shown to be a promising replacement for industrial steel fibers (ISFs) due to their reduced environmental impact and lower recycling costs [17,18,19]. Fibers are popular as one of the most feasible methods for improving the hardened qualities of ordinary cement-based composites due to their capacity to reinforce and prevent cracking. Steel fibers are one of the best and most compelling materials for boosting the flexural performance and impact resistance of concrete among commercially available fibers. The utilization of steel fibers in the construction industry necessitates vast quantities of raw materials for their industrial manufacture, resulting in serious environmental issues. In recent years, recycling, resource conservation, and sustainability have risen to the forefront of policymakers’ concerns, attracting the attention of academics in pursuit of alternate approaches to addressing the environmental consequences of the manufacture of ISFs. Recent innovations in the use of RSFs have effectively solved the challenges of the high cost, waste clearance, and environmental consequences associated with the manufacture of industrial steel fibers [17,20]. Figure 1 illustrates how well steel fibers perform in concrete.

In recent decades, the use of SFRC has steadily expanded due to its desirable features. It is presently used not just in conventional industrial and civil constructions, but also in several other fields, such as highway paving, airports, earthquake- and impact-resistant structures, bridges, and tunnels [21]. Fiber-reinforced high-strength concrete is employed not only for new constructions, but also for the repair and rehabilitation of existing structures [22]. With the fast advancement in non-destructive testing (NDT) methods, researchers now have an alternative method for evaluating the characteristics of concrete [23]. Electrical resistivity measurements can be used to analyze the distribution and orientation of fibers, and X-ray imaging can provide further assurance. The behavior of fiber-reinforced concrete parts under bending and early-age cracking in constrained fiber-reinforced mortars were both studied using acoustic emission [24,25]. Non-destructive testing for fiber-reinforced concrete is becoming more common for at least two reasons. The industry’s interest in quality management systems is a motivating element; thus, it is important to understand product quality in terms of both deviation from a quality standard and the material scattering property while producing concrete and building concrete components. The second driving factor is research, which necessitates instruments that permit viewing inside a substance as opposed to only observing its surface. This driving factor is especially powerful since global measurements of bulk attributes do not disclose the processes behind certain events. There is a need to model material characteristics on a mesoscale, as opposed to solely on a macroscale [26,27]. Numerous studies [28,29,30] that investigated the impact of fiber content on SFRC fracture characteristics have concluded that increasing the fiber volume content might significantly improve the pull-out strength and flexural strength. Li et al. [31] evaluated the flexural properties and acoustic emission behaviors of SFRC made of various fiber types (e.g., hooked-end, straight, and corrugated fibers). The hooked-end SFRC had greater acoustic emission and flexural strength values for a given fiber content than the other two SFRC varieties and plain concrete. The pull-out strengths of fibers with hooked ends buried in concrete at various angles and lengths were investigated by Cao and Yu [32]. It was found that several factors, including the fiber type, embedded inclination angle, length, and diameter, affected the peak pull-out strength of the fibers in the concrete matrix. Yoo and Banthia [33] thoroughly assessed the impact, tensile, and flexural performances of SFRC while taking the impact of the fiber orientation in the concrete into consideration. It was found that adding more fibers oriented in the direction of the tensile load would enhance the impact and tensile strength of SFRC. Using falling weights, Mindess and Zhang [34] examined the dynamic compressive failure modes of SFRC. They found that the height and mass of the falling weight had the greatest influence on the failure mode of SFRC due to the mechanical properties of the fibers. An obvious strain rate hardening effect in SFRC was discovered through several split-Hopkinson pressure bar (SHPB) studies [35,36,37]. However, Wang et al. [38] and Mansur et al. [39] found that increasing the fiber content improved concrete ductility more pronouncedly than concrete compressive strength, reducing the brittleness and improving the tensile capabilities of concrete as a result.

The proposed article begins with a discussion of the significance of steel fibers in the civil engineering industry, including their durability, impact resistance, and thermal performance. The use of industrial waste steel as fibers in concrete to address the related environmental issues is also discussed. In addition, we address the use of steel fibers in structural elements such as beams, columns, slabs, and walls, as well as the corresponding experimental parameters for flexural strength, compressive strength, and shear resistance. The use of steel fibers in various types of concrete is then presented, including self-compacting, geopolymer, pre-cast and pre-stressed concrete.

Failure mechanisms in steel-reinforced concrete are also discussed. Numerous references from recent years that demonstrate the current state of the development of SFRC were analyzed. The practical applications of steel fibers in blast loading are described, and recommendations for future research topics are provided.

## 2. Properties of Steel Fibers

The mechanical strength properties of steel fibers are useful as a primary factor for predicting the durability, fire resistance, and impact resistance of concrete. SFRC performs well in resisting tensile cracks through increased flexural strength, improving the ability of thinner structural elements to withstand heavy monotonic and cyclic loads compared to conventional concrete, regardless of its grade [40,41,42].

### 2.1. Durability

Steel fibers produce effective outcomes by enhancing the durability of concrete. Kim et al. [43] experimented with steel fibers in the marine environment to predict chloride ion penetration in the east sea of South Korea, finding improved performance and ductility in the marine environment. Papachristoforou et al. [44] experimented with cubes, slabs, and prisms with sizes of 150 × 150 × 150 mm^3^, 100 × 450 × 450 mm^3^, 150 × 150 × 550 mm^3^, and 100 ×100 × 1000 mm^3^ to test their toughness, impact resistance, freeze–thaw resistance, drying shrinkage, and abrasion resistance. The coarse aggregate was replaced with electric arc furnace (EAF) steel slag and steel fiber reinforcement in variations of around 0, 30, and 60 mm; hence, the toughness and flexural strength were enhanced in a combined effect, and the 60 mm variation provided the best results. Frazao et al. [45] compared steel-fiber-reinforced self-compacting concrete (SFRSCC) and unreinforced self-compacting concrete (SCC) by testing four beams with dimensions of 150 mm × 150 mm × 600 mm under bending load conditions. SFRSCC provided better flexural resistance and energy absorption results, though it did not improve upon the SCC properties until a load of 60 kg/m^3^ was applied. The tensile strength can be reduced at an extreme level of corrosion for SFRSCC. The various types of steel fibers, their geometric configurations, and their tensile strengths are presented in Table 1.

**Figure 2:**
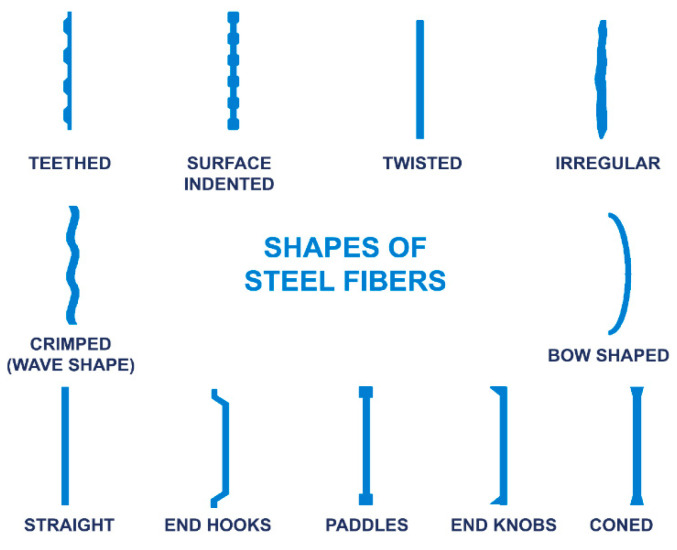
Different shapes of steel fibers.

According to Table 1, the size, shape, and carbon content of steel fibers play a significant role in their tensile strength. Macro steel fibers with a length of 13 mm and a diameter of 0.2 μm have superior tensile strength compared to other steel fibers. Due to the excess carbon in the steel, hooked-end high-carbon steel fibers with a length of 60 mm and a diameter of 0.7 μm have a high tensile strength. Consequently, if the size of a steel fiber is small, it can have high tensile strength, and if the carbon content of the steel fiber is high, it can produce better results. Marcos-Meson et al. [55] presented a literature review on the use of SFRC subjected to acid attack and found that corrosion damage may occur above a 0.3 mm crack width, leading to poor mechanical behavior. Gao et al. [46] employed recycled coarse aggregates and SFRC to conduct a test using cubes and prisms of various dimensions to anticipate freeze–thaw resistance, anti-chloride permeability carbonation resistance, and compression strength and found better durability when the water-to-cement ratio was lower. Alsaif et al. [47] used steel fibers and rubber to test five small cylinders, cubes, and prisms with sizes of 150 × 150 mm, 100 × 100 × 100 mm, and 100 × 100 × 500 mm in the marine environment and found that the best results were obtained for the 60% rubber and steel fibers, which increased the effectiveness of concrete in terms of flexibility and ductility and provided adequate distinctive transport features. Poorsaheli et al. [56] conducted a similar experiment using beams and cubes by adding polyolefin fibers and steel fibers for long-term monitoring under excessive chloride conditions. Their findings indicated a 28% increase in flexural strength and a slight decrease in corrosion resistance. Ammari et al. [57] experimented with sand concrete by incorporating steel fibers and barley straws under problematic climatic conditions. The proportions of steel fibers ranged between 0, 0.5%, 1.0%, 1.5%, and 2.0%, whereas the content of barley straws was kept constant at 15 kg/m^3^, resulting in the steel fibers showing mass variation compared with the barley straws and decreasing the porosity. Moghadam and Izadifard [48] tested both steel and glass fibers under higher temperatures ranging from 28 °C to 800 °C to predict concrete performance in terms of mechanical behavior and ductility. According to their results, steel fibers enhanced the tensile, shear, and compressive strength under excessive temperatures and demonstrated better durability. Figure 2 depicts the various steel fiber shapes that can be used in concrete.

Dezhampanah et al. [58] experimented with using waste tire rubber steel fibers in proportions ranging between 0, 0.25%, 0.5%, and 1% to test the durability parameters. Their results suggested that the 1% specimen provided better performance in terms of impact resistance, though it slightly increased the CO_2_ emissions; additionally, under the application of sulfuric acid solutions, it showed better durability. Usman et al. [59] used steel and polypropylene fibers in pre-stressed concrete girders for a real-time experiment under open-exposure conditions in Pakistan to investigate all primary durability parameters. Additionally, a scanning electron microscopy (SEM) and energy-dispersive X-ray analysis were conducted. The results showed that steel fibers provided better mechanical performance, whereas polypropylene fibers provided better durability. Abbas et al. [60] tested three different lengths (8, 12, and 16 mm) and volume proportions (1%, 3%, and 6%) of steel fibers in various concrete specimens under chloride ion penetration and mechanical degradation with different levels of chloride exposure (3.5% and 10%). The results indicated that the maximum content of steel fibers worked well, and no mechanical degradation was found. Ganta et al. [61] also experimented with SCC using both steel and glass fibers to assess the packing factor (sand-to-all-aggregate ratio), and they found that 1% was an ideal dosage for good enforcement. Nahhab and Ketab [49] used micro steel fibers coated with copper in lightweight SCC at different percentages (0.25%, 0.5%, and 0.75%), and the results showed a reduction in drying shrinkage as the fiber content increased. Afroughsabet et al. [62] tested double-hooked-end steel fibers at 1% volume in high-performance concrete with various percentages of recycled concrete aggregate and slag, which resulted in a reduction in shrinkage and water absorption.

Salmasi and Mostofinejad [63] conducted an experiment using Bacillus subtilis in lightweight aggregate concrete (LWAC) along with steel fibers under solution activities to decrease water absorption and chloride ion penetration, which occur due to minor cracks, and the results were found to be satisfactory. Afroughsabet and Ozbakkaloglu [64] tested 60 mm hooked-end steel fibers and polypropylene fibers. The volume fractions of steel fibers were 0.25%, 0.5%, 0.75%, and 1%, and the authors assessed water absorption an electrical resistivity. The results indicated better performance for the 0.85% steel with 0.15% polypropylene and reduced electrical resistivity due to the presence of the steel fibers. Karimipour et al. [65] tested steel fibers in volumes of 0%, 1%, and 2% along with rubber waste to assess drying shrinkage, the dynamic modulus of elasticity, resonance frequency, and bulk electrical resistivity; a reduction in deformation due to shrinkage was found. El-Dieb [66] utilized a unique concrete, namely ultra-high-strength self-compacting concrete (UHSC), incorporating a collection of local materials from the gulf area and steel fibers, to investigate bulk chloride diffusion, electrical resistivity, and chloride permeability under high-temperature and self-weight conditions; the results showed that the concrete was less effective under sulfate application. However, the ductility and electrical conductivity increased. Mehdipour et al. [67] tested steel fibers in concrete containing metakaolin and recycled rubber under different temperatures (150 °C, 300 °C, 450 °C, and 600 °C) for one hour. Two experiments, SEM and X-ray diffraction analysis (XRD), demonstrated that the concrete achieved better results with lower CO_2_ emissions than standard concrete. Singh [68] tested steel fibers and silica fume to assess durability.

### 2.2. Fire Resistance or Thermal Performance

Concrete should perform well against fire as it has low conductivity. Steel fibers can protect concrete from excess fire damage, and they have good thermal properties (Table 2). Serrano et al. [69] subjected steel fibers and polypropylene fibers to 400 °C thermal conditions to check the compression and spalling of concrete elements. Their findings indicated a reduction in ductility; however, the fibers provided higher compression strength. Mezzal et al. [50] tested discarded steel fibers in high-strength SCC under various temperatures (300 °C, 500 °C, and 700 °C) with a heating duration of 2 h; the fibers resulted in a two-fold increase in impact resistance and tensile strength, along with an increase in toughness and fracture energy. Fike and Kodur [70] carried out an experiment using slabs of SFRC with 63.5 mm steel fibers, supported by three unprotected steel beams and two protected girders, to test fire resistance. The results showed no need for external fire resistance measures, because the steel fibers provided good resistance against fire. Bednář et al. [71] also tested steel fibers as a replacement reinforcement in a concrete slab supported by beams under temperatures of 500 °C and 600 °C, achieved using five electrical ceramic heaters in the PAVUS laboratory in Veselí nad Lužnicí (Czech Republic); the results suggested that unique load-bearing mechanisms enhanced the steel fibers’ fire resistance, because the macrocracks formed at the level of the maximum strength when the deflection was not large enough to allow for membrane action to develop.

Jin et al. [51] tested hooked-end corrugated steel fibers in four 200 × 400 mm × 2800 mm beams in terms of fire resistance and pre-impact loading under a velocity of 5.3 m/s in order to evaluate the thermal profile, mid-span deflections, rebar deformations, and failure pattern; the finite element method (FEM) was also deployed. The fibers provided better performance when the impact load was lower. Algourdin et al. [72] experimented with cylinders and slabs incorporating steel fibers and polypropylene using a heating rate of around 10 °C/min and the ISO 834 fire standard to assess spalling sensitivity via unidirectional heat transfer and pressure measurement. Their findings showed that there were tiny pieces of surface spalling on the steel fibers cylinders. Nematzadeh et al. [73] tested steel fibers as a replacement reinforcement in recycled polyethylene terephthalate (PET) aggregate concrete in terms of fire resistance under temperatures of around 25 °C, 200 °C, 400 °C, and 600 °C. The results indicated decreased compressive strength for the same steel fibers incorporated into a steel–concrete tubular column under the same experimental temperatures.

### 2.3. Impact Resistance

**Figure 3:**
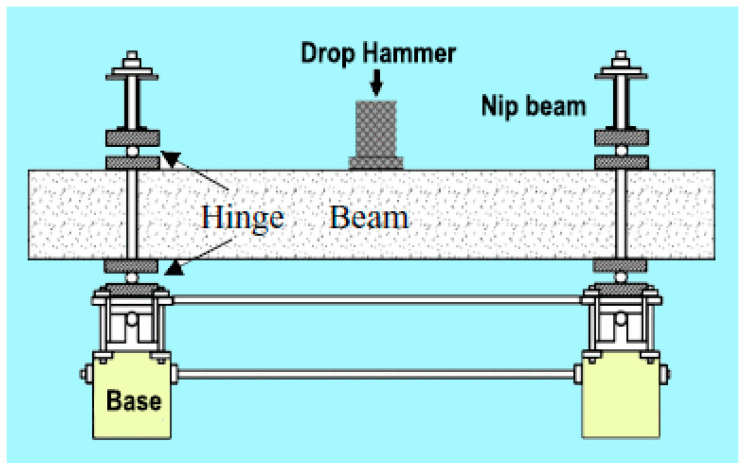
Drop hammer experiment process.

Impact resistance is defined as the ability of concrete to resist frequent blows without spalling and cracking [74]. Choudhary et al. [75] experimented with SFRC wall panels containing various percentages of hooked-end steel fibers, as well as bare concrete, under drop weight impact loading with a 4.5 kg mass and a falling height of 450 mm. Their findings suggested that a greater quantity of steel fibers provided better impact resistance. Song et al. [76] used concrete cylinders for experiments with and without steel fibers under a drop weight test. The steel fibers were long and had hooked ends, and the concrete was mixed with crust stone silica fume. The high-strength steel-fiber-reinforced concrete (HSFRC) achieved the best results, and the initial cracks and failure strength were found to be around 3.9-times and 4.2-times better, respectively, than those of high-strength concrete. Nili and Afroughsabet [52] tested steel fibers (hooked-end, with a length of 60 mm and an aspect ratio of 80) and silica fume in all three significant concrete specimen shapes (cubes, cylinders, and prisms). An impact test was carried out with a 4.45 kg hammer and a drop of 457 mm for the steel fibers and silica fume, which demonstrated a better impact resistance and an enhanced ability to absorb kinetic energy and arrest crack propagations. Figure 3 depicts the experimental process of the drop hammer test for impact resistance.

Kim and Yoo [77] investigated three different steel fibers (half-hooked, straight, and twisted) at two different angles (0 °C and 45 °C) under two different temperatures (ambient and a cryogenic temperature of −170°C), subjecting the specimens to impact and static loading conditions. Specimens with a cross-sectional area of 25 × 25 mm subjected to pull-out tests showed increasing stiffness for inclined steel fibers under an ambient temperature; however, for the cryogenic temperature, the outcomes were marginal. Vivas et al. [78] assessed three different fibers, namely steel, polymer, and glass, using a drop weight impact test with a 5 kg mass and a 150 mm height and a static residual capacity test. The comparison study showed improvements in the cracking and post-cracking phases for the steel fibers, and increased toughness was found for the steel and polymer combination.

Yoo et al. [79] experimented with pre-stressed concrete (PSC) sleepers 2400 mm in length containing steel fibers and ground granulated blast furnace slag (GGBFS) under impact loading using two hammers with weights of around 400 and 500 kg and a 2 m fall height, resulting in superior performance for the 0.75% variation hooked steel fibers. Shin et al. [80] subjected PSC sleepers of the same dimensions to multiple impacts, producing similar outcomes. Reddy and Srinivasa Rao [81] evaluated crimped-type steel fibers (30 mm length, 60 aspect ratio) in cylindrical-disk specimens of a unique concrete called ternary concrete (TC) along with regular concrete using an impact drop test with a 44.5 N hammer and a height of 457 mm. The fibers increased the test outcome by several blows, and improved results were found for the fibrous TC. Prasad and Murali [82] assessed 180 cylindrical disks (76 mm radius, 64 mm thickness) containing various dosages of steel fibers (including two functionally graded specimens) via a falling mass impact test with a mass of 4.54 kg and a height of 457 mm, demonstrating satisfactory results for single-layer fibrous concrete under compression. Table 3 displays the majority of research investigating the impact resistance properties of steel fibers.

Mahakavi and Chithra [83] evaluated two different steel fibers, crimped and hooked, in SCC via impact loading with a 4.5 kg ball hammer and a falling height of 450 mm for cube, prism, and cylindrical disk specimens. The findings suggested that the concrete with hybrid hooked-end fibers withstood the maximum impact, with increased compression strength. Islam et al. [84] tested steel fibers in lightweight geopolymer concrete panels (600 × 600 × 50 mm), which consisted of GGBS and palm oil fuel ash, under a drop hammer test from a height of 300 mm with a 10 kg weight; at 0.5% content, the fibers increased the duration of the initial crack stage, achieving a small crack width and high impact resistance. Abid et al. [85] carried out repeated drop weight tests with weights of 4.5, 6, and 7.5 kg and heights of 450, 575, and 700 mm on SCC containing various percentages of micro steel fibers and showed better performance for the 4.5 kg/450 mm test along with an improvement in the impact ductility of around 24%. Nataraja et al. [86] assessed different proportions of steel fibers in standard concrete under a drop weight test, and the fibers resulted in better performance. Li et al. [87] experimented on two different sizes of coarse aggregates (8 and 25 mm) with three different steel fibers (30 mm medium hooked-end, 60 mm long 5D, and 13 mm short straight) using a pendulum impact test with a mass of 22 to 40 kg and a height of 0 to 4 m; the results showed that the 30 mm medium hooked-end and 60 mm long 5D fibers were effective under impact.

## 3. Application of Steel Fibers in Structural Elements

Steel fibers can be utilized in structural elements to draw a strength comparison with conventional concrete. This section discusses the implementation of steel fibers in the following structural elements to assess performance via shear, compression, and tension tests: beams, columns, slabs, and walls.

### 3.1. Beams

Experimental studies have been published in the literature investigating the flexural and shear strengths of reinforced concrete (RC) beams containing steel fibers. The results have shown that steel fibers can be adopted to enhance both flexural and shear strength.

#### 3.1.1. Flexure

Flexure is a primary parameter of beams for predicting concrete behavior under tension. Karimipour and Ghalehnovi [88] utilized steel and polypropylene fibers in 0, 1%, and 2% volume variations in RCA concrete to experiment on 54 RC beams with dimensions of 150 × 200 × 1500 mm under four-point loading; the fibers improved the results for maximum deformation, and the flexural strength increased moderately. Rashid et al. [89] experimented with 15 RC geopolymer concrete beams with dimensions of 100 × 200 × 1500 mm containing different types of rebar (steel-fiber-reinforced polymer (FRP), carbon FRP, and basalt FRP bars), which improved both tension and compression strength by around 8 and 6 mm, respectively. The findings suggested that the steel-FRP provided more promising outcomes in terms of cracking parameters compared to the other FRP types. Yoo and Moon [90] evaluated hooked-end steel fibers in RC beams with four different reinforcement ratios under a four-point bending experiment. The results showed an increase in flexural strength when the percentage of reinforcement increased. Jafarzadeh and Nematzadeh [91] assessed glass FRP bars and steel fibers in 11 RC beams via a flexural four-point bending test under high temperatures of 20 °C, 250 °C, 400 °C, and 600 °C. An analytical model was developed using the sectional analysis method. The findings indicated an increase in flexural capacity up to 400 °C and a reduction at 600 °C.

**Figure 4:**
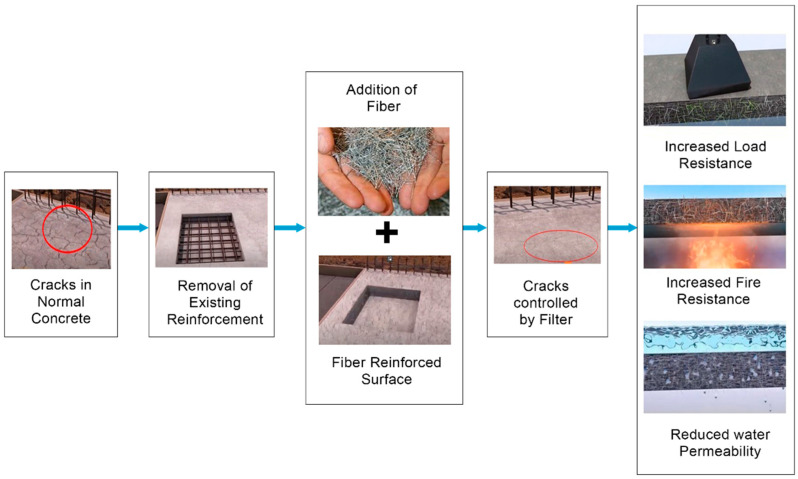
Mechanism for the repair of cracks in SFRC.

Yoo and Choo [92] experimented with slabs of two thicknesses (50 mm and 100 mm) containing steel fibers supported by an inverted T-steel girder via a three-point loading test and an analytical model, and the outcomes were better for the steel fibers in the thickest slab. Tran et al. [93] conducted both experiments and analyses on steel fibers of varied lengths and volume fractions in geopolymer concrete (GPC) beams subjected to a four-point bending test, showing enhanced outcomes. Mertol et al. [94] compared a 3.5 m beam containing steel fibers and various longitudinal reinforcements to a conventional RC beam under four-point bending, and satisfactory results were obtained for the SFRC beam. El-Sayed [95] conducted a comparison experiment and developed a non-linear FEM for beams containing lathe waste and steel fibers and regular concrete beams under two-point loading conditions. The findings indicated that lathe waste could be a suitable alternative to steel fibers in terms of flexure. Mahmod et al. [96] tested steel fibers in SCC under four-point loading, finding an increase in flexural strength; furthermore, the outcomes were compared with theoretical results based on the American Concrete Institute (ACI) 318 code. The mechanism of crack healing in SFRC is depicted in Figure 4.

Sarmiento et al. [97] examined the distribution and orientations of steel and polymer fibers in RC beams in both lab and analytical models under three-point loading to aseess the flexural capacity, and the results showed that the polymer fibers were more evely distributed that the steel fibers. Meng et al. [98] developed a simulation model to predict the bending performance of steel fibers across four stages (the initial cracking stage, cracking moment, crack development stage, and failure stage), and it produced acceptable outcomes for the bending deformation characteristics of SFRC according to a comparison with the data of several experiments. Zhu et al. [99] subjected SFRC beams containing FRP bars to a four-point bending test with repeated loading to assess the flexural capacity. The findings suggested that an increase in the quantity of steel fibers produced promising outcomes. Sivakamasundari et al. [100] conducted an experiment on beams with and without steel fibers (with biaxial geogrids replacing shear reinforcements) subjected to three-point loading, and they obtained outstanding results for specimens containing biaxial geogrids with steel fibers in terms of flexure. Berrocal et al. [101] monitored cracks in RC beams with and without steel fibers under corrosion conditions for around three years; then, they subjected the beams to a three-point loading test, finding 1 Mpa reduction in flexure due to corrosion.

#### 3.1.2. Shear

Shear tests in beams assess the shear strain, shear stress, and shear modulus. They help to predict beam failure under shear. Algassem et al. [102] tested 125 × 250 × 2440 mm RC beams containing different types and volumes of steel fibers (including various combinations of stirrups) under blast and static loading using a shock tube and four-point loading test, respectively. The fibers acted as an alternative to stirrups during blast loading, resisting shear and providing better outcomes at a 1% volume proportion. Tahenni et al. [103] evaluated a 100 × 150 × 900 mm beam containing steel fibers with and without stirrups under two-point loading, finding similar results for both specimens. Yoo et al. [104] investigated steel fibers with and without stirrups in three types of beam under four-point bending, and the findings indicated the lowest possible number of stirrups that could be used in addition to steel fibers for beams of limited size.

Joshi et al. [105] applied a four-point bending experiment to concrete containing two different fibers, namely steel fibers and macro synthetic structural polyolefin fibers, in various volume proportions; the 0.35% volume variation limited shear cracks. Zhang et al. [106] experimented with the numerical modeling of an SFRC slab and a composite steel beam linked by a shear connector, which showed effectiveness in resisting shear cracks. Chidambaram and Agarwal [107] conducted laboratory experiments on beams containing geogrids and steel fibers under three-point loading, showing enhanced shear capacity. Shahnewaz and Alam [108] investigated shear capacity parameters such as the aspect ratio of fibers, fiber volume, the longitudinal reinforcement ratio, the compressive strength of the concrete, and the shear span-to-depth ratio via the metamodel of optimal prognosis (MOP), forming the equation using genetic algorithms. Gali and Subramaniam [109] predicted cracks using two fractions of steel fibers (0.5% and 0.75%) under three-point loading by employing the digital image correlation (DIC) technique. The findings indicated that a fiber content of 0.75% enhanced the resistance capability of the beams. Lakavath et al. [110] carried out a similar experiment specifically for pre-stressed concrete beams with steel fiber content variations of 0.5% and 1%, resulting in a reduction in the opening of cracks. Chaboki et al. [111] tested various volumes of recycled aggregate and steel fibers in RC beams incorporating stirrups with divergent spacing. The results showed an increase in maximum strain and shear behavior.

Ismail and Hassan [112] evaluated various volumes of steel fibers and crumbed rubber. Self-consolidated vibrating concrete was used under four-point loading and demonstrated good shear resistance. Ding et al. [113] assessed the combined effect of steel fibers and stirrups in self-consolidated vibrating concrete, finding an increase in shear loading [114]. Zamri et al. [115] experimented with short-recess (SR) and deep-recess (DR) pre-cast half-joint beams containing steel fibers, observing an enhancement in shear strength. Conforti et al. [116] subjected RC wide–shallow beams (WSBs) containing steel fibers to four-point loading and found enhanced shear strength.

### 3.2. Columns

Steel fibers enhance the performance of columns under compression, and the addition of various volume percentages of steel fibers helps assess the behavior of columns under different loading conditions.

#### Compression

A compression test, i.e., the application of compressive force, is the best and easiest way to predict a concrete’s health status. Atea [117] tested the combined effect of five short, square RC columns (100 × 100 × 1000 mm) containing steel and polypropylene fibers under axial compression. The experiment considered variations in lateral tie spacing in addition to longitudinal reinforcement. Two aspect ratios of steel fibers were included (100 and 60), and an FEM was developed. The study concluded that the compressive strength was moderately increased. Usman et al. [118] tested 150 mm diameter cylinders containing three varieties of wrapped steel fibers (steel pipe confinement, carbon-fiber-reinforced polymer (CFRP) sheet, and conventional) under axial compression. The results suggested that the incorporation of steel pipe confinement and CFRP had a favorable effect on the compressive strength and lowered the brittleness to a certain extent. Li et al. [119] experimented with a concrete-filled steel tube (CFST) column containing various volume percentages of steel fibers and SCC, assessing self-stress and steel tube thickness under axial compression; the fibers enhanced the ultimate load along with the post-peak behavior. Xu et al. [120] tested ultra-high performance (UHP) concrete in CFST stub columns with various percentages and aspect ratios of steel and polypropylene fibers and different steel tube thicknesses under axial compression, and the steel fibers provided satisfactory outcomes. Liu et al. [121] evaluated self-stressing RCA concrete in CFST columns containing steel fibers under axial compression, finding enhanced ductility; however, the compression results were less favorable.

Jang and Yun [122] experimented with cylinder and prism specimens containing steel fibers and various coarse aggregates subjected to a standard compressive strength of 60 N/mm^2^, observing better compressive strength performance. Nematzadeh et al. [123] experimented on and developed a model of 18 RC square columns containing steel fibers and CFRP wraps under eccentric loading conditions. They included three different eccentricity ratios (0.29, 0.46, and 0.63), which resulted in less effective steel fibers; however, enhanced ductility was observed. Park et al. [124] assessed column orientation and the distribution of steel fibers in real-time specimens employing a micro-computed tomography (CT) scanning instrument, and they found orientations at the bottom perpendicular to the primary axis, which were less beneficial to the compressive strength. Nematzadeh et al. [125] investigated steel fibers and tire rubber in CFST stub columns exposed to high temperatures of 20 °C, 250 °C, 500 °C, and 750 °C under axial compression. The findings indicated that the strength remained constant until 500 °C, and a 34% reduction was found around 750 °C. Savino et al. [126] developed a numerical model of specimens containing steel fibers of various types, volumes, and aspect ratios to predict compressive strength results, finding a possible 2% to 36% compression strength enhancement.

### 3.3. Walls

Steel fibers are essential ingredients for wall structures, as they enhance strength and durability. Zhao and Dun [127] developed a shear wall with a height of 1800 mm, length of 900 mm, and thickness of 200 mm supported at the bottom by a base girder and at the top by a beam containing various volumes of shear-cut steel fibers and subjected it to a constant axial load. They applied a horizontal reverse cyclic load to assess steel strain, concrete strain, crack load, lateral displacement, and ultimate load. Hysteretic loops and skeleton curves were created to evaluate the ultimate load and cracking load point. Carrillo et al. [128] conducted a shake table experiment on walls containing different dosages of steel fibers with aspect ratios 64 and 80, aiming to reach maximum load in order to determine the failure mode, crack pattern, and hysteretic response lateral resistance; they found that SFRC performed remarkably well.

Petkune et al. [129] carried out a two-phase experiment on six steel-framed shear walls containing hybrid steel FRPs (including carbon FRPs and glass FRPs). Phase one of the experiment involved the subjection of a pristine specimen to quasi-static cyclic loading based on the ATC-24 protocol, and phase two assessed self-induced damage for retrofitting, which demonstrated a higher response than the pristine specimen. Holden et al. [130] evaluated steel fibers in precast cantilever concrete wall panels 4000 mm heigh, 1350 mm long, and 125 mm wide. The wall panels containing post-tensioned unbonded carbon fiber tendons were divided into two categories, pre-cast and partial pre-stressed, and they were subjected to quasi-static reverse loading.

### 3.4. Slabs

Steel fibers are becoming a vital material for application around the edges and joints of slabs to resist cracks. Soltani et al. [131] experimented on and created an FEM for 14 slabs with dimensions around 1000 × 1000 × 75 mm divided into four varieties (conventional, excluding reinforcement, externally bonded GFRP sheet, and steel-fiber-reinforced). Under dynamic loading conditions achieved via a drop weight test, the steel fibers showed acceptable performance compared with the conventional and excluding reinforcement specimens; however, the externally bonded GFRP sheet provided superior outcomes. Nguyen et al. [132] investigated 0.51% and 0.89% volume variations of steel fibers and 0.11 and 0.22 variations of polypropylene webs. Straight and hooked steel fibers were incorporated into pre-cast/pre-stressed concrete hollow-core (PCHC) slabs with dimensions of 4150 × 1200 × 400 mm and four non-circular voids, and the specimens were subjected to unsymmetrical line loading under shear behavior. The findings suggested that the 0.89% volume fraction straight steel fibers produced remarkable outcomes. Hoang [133] created a model using the artificial neural network (ANN) and piecewise multiple linear regression (PMLR) approaches for 140 specimens to predict the punching shear capacity of an SFRC flat slab, and the sequential PMLR showed excellent performance. Peng et al. [134] developed a 3D mesoscale model of a UHP-SFRC slab to anticipate the slab thickness and crater damage. Kueres et al. [135] examined punch shear capacity by developing a theoretical model of an RC slab containing different volumes of steel fibers, and the results were compared with previous experimental data for validation.

## 4. Application of Steel Fibers in Different Types of Concrete

Alongside incorporating steel fibers into conventional concrete, they would be a beneficial addition to other types of concrete, such as pre-stressed concrete, pre-cast concrete, self-compacting concrete, and geopolymer concrete.

### 4.1. Pre-Stressed Concrete

**Figure 5:**
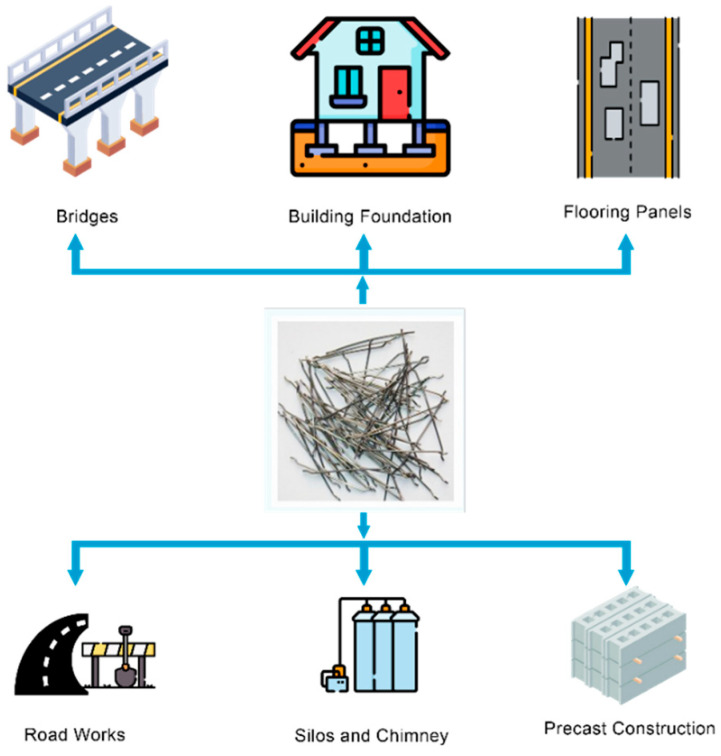
Utilization of steel fibers in various construction practices.

Pre-stressed concrete improves the tensile properties of structural elements; hence, the addition of steel fibers can further enhance its strength and durability. Paramasivam et al. [136] compared experimental outcomes with an FEM of eight moderately pre-stressed T-steel fiber concrete beams under shear behavior. The experiment considered several variables: the volume fraction, the partial ratio of pre-stressing, and the shear span-to-sufficient-depth ratio. The beams were entirely supported with an adequate depth of 185 mm under two-point loading. The results showed a 40% rise in ultimate strength with a 1% proportion of steel fibers, and the experimental results matched the FEM values. Liu et al. [137] compared the non-linear structural behavior of three ratios of SFRC and high-strength concrete in pre-stressed concrete beams with box sections. The results showed an increase in crack behavior and structural stiffness when steel fibers were included. Figure 5 depicts the utilization of steel fibers in several different construction practices.

Narayanan et al. [138] incorporated steel fibers as a web reinforcement in seven rectangular variations of simply supported RC beams with dimensions of 85 × 150 mm to assess shear strength under symmetrical four-point loading. The performance remained similar for the steel fiber variations. Akhnoukh and Xie [139] also investigated steel fibers. They incorporated welded wire reinforcement (WWR) (grade 18) into pre-cast/pre-stressed UHP concrete girders (for implementation in bridges according to standard guidelines), namely American Association of State Highway and Transportation Officials (AASHTO) type-2 girders, to compare the economic feasibility and structural efficiency under shear stress with four-point loading. WWR provided significantly improved results compared to steel fibers.

### 4.2. Pre-Cast Concrete

Pre-cast concrete containing steel fibers improves structural performance in terms of cracks and ductility. Mohamed and Nehdi [140] developed an FEM of pre-cast SFRC pipes using ABAQUS software and previously reported data from tests involving three edge-bearing methods to predict the optimized dosage and type of steel fibers, in addition to creating a design tool; the model’s average prediction error for ultimate D-loads was around 6.5%. Turmo et al. [141] experimented on steel-fiber-reinforced concrete panels located in dry castellated joints in a segmental bridge, subjecting them to shear capacity tests (including a cohesion test, friction test, and combined test) with open and closed joints and various pre-stressing ratios; they found a decreased effectiveness for panel joints. Zamri et al. [142] investigated SR and DR half-joint SCC beams containing a 1% volume of steel fibers, subjecting them to shear load with a shear span-to-depth ratio of 1.4 and 2.1. The experimental results were compared with previously reported theoretical predictions based on analytical and RILEM methods, which were shown to be approximate. The findings indicated that a 1% steel fiber volume achieved effective outcomes, demonstrating the possibility of replacing 50% of the horizontal and vertical reinforcement and enhancing the tensile resistance.

### 4.3. Self-Compacting Concrete

**Figure 6:**
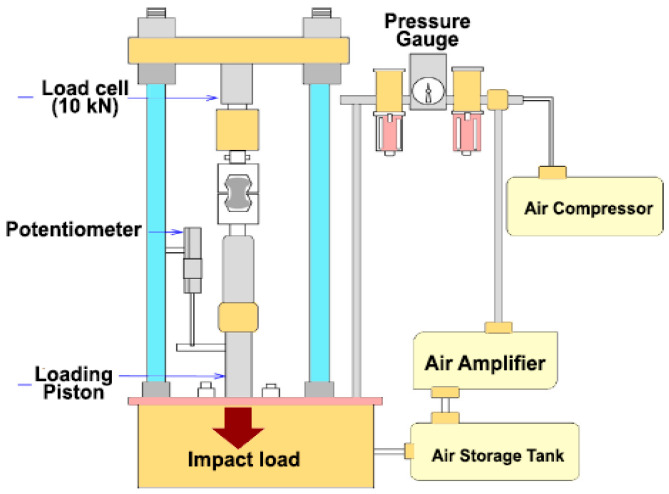
Pull-out test experiment setup.

Majain et al. [143] investigated nine 200 × 200 × 200 mm SCC cubes containing 3.5 cm long hooked-end steel fibers with an aspect ratio of 63.6 and ribbed bars with diameters of 12, 16, and 20 mm using a direct pull-out test to assess the concrete bonding behavior. The authors observed outstanding outcomes in terms of enhances tensile and compressive strength and improved bonding. Li et al. [144] created an ideal mix design using a new approach known as paste film thickness theory and a combination method to experiment with steel-fiber-reinforced self-compacting lightweight aggregate concrete (SFSLC) and assess workability along with other mechanical properties. The results showed decreased filling and passing ability and a rise in segregations; furthermore, micro steel fibers provided better outcomes than long steel fibers in terms of mechanical behavior. Sanjeev and Sai Nitesh [145] found this material less effective in terms of workability; however, it displayed enhanced mechanical properties, such as flexural and spilled tensile strength. The pull-out test experimental set up is depicted in Figure 6.

### 4.4. Geopolymer Concrete

Due to the high environmental impact of conventional concrete production, the development of geopolymer concrete is considered the most important innovation in the construction sector [146]. The addition of fly ash or granulated blast-furnace slag into concrete leads to environmental advantages such as reducing CO_2_ emissions and increased waste reuse [147].

The current literature still has gaps related to research on the effect of steel fibers on parameters such as setting time, thermal conductivity and resistance, abrasion resistance, carbonation resistance, freeze/thaw resistance, corrosion resistance, electrical resistance, electrical resistivity, sorptivity, and chemical and electrical resistance [148]. Nevertheless, studies have been conducted demonstrating that steel fibers help to increase both the compressive and tensile behavior of geopolymer concrete and achieve good workability. Liu et al. [149] tested ultra-high performance geopolymer concrete (UHPGC) containing various categories of steel fibers (four straight and two deformed shapes with aspect ratios of 50, 67, 108, 65 and 65, 65, respectively) in terms of flexural behavior, compressive strength, flowability, and energy absorption capacity. The results suggested that when the fiber content increased and the diameter decreased, the mechanical properties and flexural strength increased.

Liu et al. [150] experimented with three variations of geopolymer-based ultra-high-performance concrete (G-UHPC) (steel-fiber-reinforced, ceramic ball aggregate, and plain) under projectile penetration to assess projectile impact resistance in terms of crater damage, the depth of penetrations, and crack propagation. An FEM was developed using LS-DYNA software to validate the experimental results. The results concluded that the combination of ceramic ball aggregate with steel fibers showed remarkable outcomes. Liu et al. [151] experimented with steel fibers and silica fume in UHPGC to assess fracture and mechanical properties, producing satisfactory results. Meng et al. [152] carried out an experiment on alkali-activated steel-fiber-reinforced geopolymer involving a methane gas explosive test using portions of a slab extracted from a tunnel with dimensions of 1800 × 400 × 90 mm and 12,000 × 1800 × 600 mm, resulting outstanding resistance. Zhang et al. [153] experimented with introducing volumes of milling steel fibers ranging between 0 and 2.5% into geopolymer concrete. The results proved that when the steel fiber content was below 2%, the cubic and axial compression strength increased, but when the boundary of 2% was crossed, these parameters decreased.

## 5. Blast Loading or Blast Resistance

Blast-resistant concrete provides more flexibility to ensure safety against blasts; however, steel fibers enhance the strength of concrete with or without reinforcement conditions. Foglar et al. [154] experimented with a combination of waste steel fibers, which contributed to a lower ductility, and a substantial amount of polypropylene fibers to assess blast performance in an RC pre-cast slab with dimensions of 300 × 1500 × 6000 mm. To perform the blast experiment, a 25 kg TNT charge was produced in the center of the slab, and numerical simulations were also developed; a moderate effect of waste steel fibers on blast performance was found. Li et al. [155] presented a comparative study on various fibers incorporated into RC slabs (2000 × 800 × 1200 mm), such as steel fibers, steel wire meshes, and ultra-high-molecular-weight polyethylene (UHMWPE) fibers, under blast loading with the application of TNT charges of around 6, 8, and 12 kg from 1500 mm above the middle portions of the slab. The results suggested that the hybrid slab produced superior outcomes.

Luccioni et al. [53] experimented with high-carbon 60 mm long hooked-end steel fibers with a high compression strength of around 114 N/mm^2^ to assess blast response via the shock-wave method for an RC square slab with dimensions of 550 × 50 mm. A cylindrical shock wave was created with a gel-like explosive, and the steel fibers proved effective by improving the blast behavior; the authors implemented a similar experiment with various sizes of hooked-end fibers, and the numerical simulation obtained more effective results for shorter fibers [156]. Li and Aoude [54] tested the incorporation of steel fibers into HSC with two grades of high-strength steel reinforcement (690 and 400 N/mm^2^) under a shock tube experiment at the University of Ottawa (Canada). A 2D FEM was also developed, and the results showed that the steel fibers were effective for improving blast resistance in terms of controlling damage and reducing displacement. Gong et al. [157] presented a review on ultra-high performance concrete using steel fibers.

## 6. Corrosion Effect on Steel Fibers

SFRC increases resistance to crack propagation and permeability, which are primary factors for increasing corrosion; the inclusion of anti-corrosion chemicals in steel fibers helps to resist corrosion [158]. Tang and Wilkinson [159] experimented with SEM and energy-dispersive X-ray spectroscopy (EDS) for a railway tunnel structure. The research’s ultimate motive was to determine the corrosion activity of SFRC under a stray alternating current (AC). They included low-carbon steel fibers around 0.75 mm in diameter and 62 mm long; the findings indicated adequate corrosion resistance in the absence of a chloride environment. Tran et al. [160] adopted two different steel fibers (twisted and hooked) in strain-hardening steel-fiber-reinforced cementitious composites (SH-SFRCs); high-strength mortar was also used. All the specimens were dipped in 3.5% chloride before testing their direct tensile strength, as well as calcium nitrate, which provided superior resistance against corrosion.

Liu et al. [161] applied a zinc phosphate conversion coating to steel fibers and test them via SEM and XRD under high temperatures to assess corrosion. The fibers showed effective results up to 75 °C; however, they became less effective when the temperature rose beyond 85 °C. Fan et al. [162] experimented with UHPC containing various amounts of steel fibers and lightweight sand (LWS) under linear polarization resistance (LPR), electrochemical impedance spectroscopy (EIS), and potential (OCP) Tafel polarization, involving open-circuit and electrochemical tests. The results were remarkable for a volume of 25% LWS and 3% steel fibers [163,164]. Wang and Niu [165] incorporated steel fibers into shotcrete to test for sulfate corrosion resistance and obtained satisfactory results.

## 7. Failure Mechanisms in Concrete with Steel Fibers

Failure scenarios in concrete incorporating steel fibers must be observed to provide adequate structure safety. Tóth et al. [166] experimented with predicting the failure and strength of steel fiber anchorages. Consequently, 62 shear-loaded and pull-out tests were conducted on shear-loaded steel anchors in plain concrete (PC) and SFRC. According to the test results, SFRC exhibited superior load-displacement behavior to PC. The SFRC increased the ductility of the fastening systems and could withstand more failure scenarios in concrete than PC [166]. Lin et al. [167] experimentally and analytically investigated the failure mechanisms of S-UHPC. Five push-out tests were conducted to compare the functional performance of steel plates and UHPC.

To evaluate the structural behavior of S-UHPC composites, static experiments were conducted, and a finite element (FE) model was developed and validated based on the experimental results. In addition, a parametric study was conducted to summarize the failure modes of the push-out test and investigate the mechanism of steel fibers’ influence on the structural behavior of S-UHPC composite beams. In conclusion, an analytical model for predicting the ultimate strength of S-UHPC composite beams was proposed and compared with existing design codes. The outcome was positive and advantageous [167]. High-performance steel-fiber-reinforced concrete (HPSFRC) was utilized by Li et al. [168] to investigate the dynamic mechanical properties of the materials. Fiber contents of 0.1% and 2% were chosen for the experiment. LS-DYNA was used to simulate the dynamic failure process of HPSFRC, and failure patterns with varying fiber contents were compared. The energy dissipation ratio rose with an increasing steel fiber content and fell with an increasing strain rate. The steel fibers enhanced the tensile strength of the HPC under quasi-static rather than dynamic loading [168]. Dalvand et al. [169] examined the effects of partially substituting Portland cement with pozzolanic material on the fresh and hardened properties of high-strength self-compacting (HSSC) cementitious composite reinforced by randomly distributed wavy steel fibers. They examined various volume fractions of wavy fibers (0.25%, 0.50%, 0.75%, and 1%) and silica fume contents of 5% and 15% cement. The tensile strength, water absorption, flexural strength, compressive strength, and impact resistance of fifteen different mixtures were evaluated. Utilizing X-ray microanalysis (EDX), the chemical compositions of the cementitious matrix were quantified. This study’s findings improved our understanding of the failure mechanisms of self-compacting fibrous composites [169]. Zhang et al. [170] simulated the dynamic failure behavior of SFRC at room temperature and elevated temperatures using a three-dimensional mesoscale model. The simulation results indicated that at 2% volume, the effect of steel fibers on the thermal conductivity of SFRC could be ignored. The compressive strength of SFRC was less sensitive to the strain rate than that of ordinary concrete, and it decreased at high temperatures. Regardless of the temperature, the performance of steel fibers in SFRC increased with an increase in the fiber volume fraction and decreased with an increase in the strain rate. Increasing the fiber diameter or length while maintaining the same number of fibers decreased the dynamic compressive strength of the SFRC [170].

## 8. Comparison of SFRC Studies

Decades of research have been conducted on steel fibers in structural applications, and as a result, steel fibers have become a popular research topic in civil engineering. Researchers are currently exploring several innovative ideas to make the best use of steel fibers in concrete. In this article, we discussed numerous previously published, high-quality works on SFRC; we will now compare their findings.

1. The presence of steel fibers in concrete enhances mechanical properties such as flexural strength, compression, and splitting tensile strength. However, the flexural strength improves significantly more than the compression and splitting tensile strength, so SFRC is superior to conventional concrete.
2. SFRC provides superior resistance to cracking compared to conventional concrete; however, the steel fibers must be evenly distributed in the concrete without any gaps, and a vibrating machine is essential for SFRC to achieve the desired results.
3. Due to their superior resistance, strength, and flowability, hooked, straight, crimped, and twisted steel fibers are more commonly used than other forms, allowing for their easy distribution across entire portions of concrete.
4. Hooked-end steel fibers provide greater efficiency compared to other forms of steel fibers due to the presence of strong, secure elements, and they can produce greater pull-out resistance; therefore, they can be used in structural applications to improve the performance of concrete.
5. SFRC provides superior fire resistance properties compared to conventional concrete, thereby enhancing the durability to some degree, and it also consistently outperforms conventional concrete under blast loading conditions, thereby aiding in the protection of structures from such impact or dynamic loading conditions.
6. Steel fibers cannot serve the same purpose as primary reinforcements, so rebars should be considered as the primary source of reinforcement in concrete. Steel fibers are used to stop cracks from spreading, because rebars cannot stop cracking. Consequently, by adding additional support, steel fibers can be used to increase the strength of reinforced concrete.
7. If steel fibers are used in combination with rebars, the dimensions of the structural members could be limited, because steel fibers improve resistance to dynamic and impact loads and resist material disintegration, thereby eliminating the need to design beams with large dimensions.

## 9. Future Scope and Recommendations

We recommend that the future scope of SFRC research should focus on enhancing the system’s overall performance and efficiency to obtain improved data. The predicted areas for future progress are listed below [37,55,71,103,107,124,128,159].

- Further research is necessary regarding a more effective experimental process to assess the fire resistance of SFRC; hence, heavily damaged beams should be used for experiments to predict optimal resistance against fire.
- Dynamic pull-out experiments should be performed to evaluate the dynamic behavior of UHPC, and advanced test machines should be used for impact tests incorporating comparisons with various types of steel fibers.
- Steel fibers are often improperly distributed and poorly orientated in concrete; hence, several parameters, such as casting length, mixture viscosity, flowability, and casting height, should be considered to optimize their distribution.
- Both AC and DC conditions should be implemented in electric railway tunnels for future research on corrosion resistance.
- Experiments involving shotcrete should be improved by using various fly ash and steel fiber content proportions and water/binder ratios, and durability can be analyzed through multifactor coupling.
- To assess the fiber–matrix bond under acid attack, reactive mass transport modeling must be considered. Several experimental tests with varying loads and types of steel fibers should be developed.

## 10. Conclusions

The current article provided a review of the properties and applications of steel fibers in civil engineering. The best implementation strategies for enhancing steel fiber usage were discussed by summarizing the fundamental guidelines and techniques extracted from previously published peer-reviewed studies. The primary conclusions from this review article are provided below.

- The article highlighted the significance of steel fibers in the construction industry according to previously reported studies. The most detailed and insightful studies were presented, along with detailed experimental data, including comparisons of RC structures with and without steel fibers.
- Steel fiber properties such as impact resistance, durability, and fire resistance were presented. Experiments such as chloride ion penetration tests, ultrasonic pulse velocity tests, compression tests, and abrasion resistance tests were also discussed.
- In this study, the critical performance of SFRC was thoroughly evaluated using both experimental and analytical data. In addition to related parameters such as shear and compression strength, the methodologies applied to evaluate the use of concrete containing steel fibers for primary structural elements such as beams, columns, slabs, and walls were discussed.
- The various shapes and volumes of steel fibers used in SFRC as well as their application in various types of concrete, such as geopolymers, SCC, and pre-cast and pre-stressed concrete, were reviewed. This article also discussed the operational evaluation and performance of SFRC under blast loading, as well as its performance under corrosive conditions.
- After reviewing previous articles, we concluded that SFRC can be used to produce conventional RC members with higher impact resistance, boosting their resistance to local damage and spalling. SFRC can limit fracture development and crack widening, supporting the use of high-strength steel bars without excessive crack width or deformation under service loads.
- SFRC increases the ductility of conventional RC elements, hence enhancing their dynamic stability and structural integrity. SFRC also enhances the shear resistance of RC members. As a result, failure due to slab punching shear can be minimized.
- After a thorough review of the literature, we concluded that steel fibers can be used in flat slab structures for punching shear resistance and military structures for resistance against blasts or impact loading in addition to conventional rebars. Steel fibers may also be used in temporary structures such as drain slabs without rebars, but care must be taken to ensure that there will be less load impact.
- Due to their superior resistance to cracking and dynamic loading, SFRC structures are more expensive to build than conventional concrete structures, but their long-term maintenance costs are nearly equivalent.

## Figures and Tables

**Table 1:** Various steel fiber types, including their geometry and tensile strength.

Authors, Year, and Reference Number	Fiber Types and Shapes	Diameter (μm)	Length (mm)	Tensile Strength (MPa)	Experiment’s Primary Objective
Danying Gao et al. (2020) [46]	Hooked at both ends	0.6	30.5	1000	Durability
Abdulaziz Alsaif et al. (2018) [47]	Manufactured steel fibers (MSFs)	0.8	55	1100	Durability
	Screened recycled tire steel fibers (RTSFs)	<0.3	15–45	2000	Durability
Moghadam et al. (2020) [48]	Single-hooked-end fibers	0.8	30	1150	Durability
Nahhab et al. (2020) [49]	Micro steel fibers	0.2	13	2600	Durability
Mezzal et al. (2020) [50]	Discarded steel fibers (DSFs) (straight)	0.92	10–35	1400	Thermal performance
Jin et al. (2018) [51]	Hooked-end corrugated fibers	0.6	30	1100	Thermal performance
Nili et al. (2010) [52]	Hooked-end steel fibers	0.75	60	1050	Impact resistance
Luccioni et al. (2017) [53]	Hooked-end high-carbon steel fibers	0.7	60	2300	Blast resistance
Yang Li et al. (2020) [54]	Hooked-end steel fibers	0.55	30	1350	Blast resistance

**Table 2:** The majority of experiments on the fire-resistant properties of steel fibers.

Authors and Year	Type and Size (mm) of Specimens	Experiments Conducted
Ruben et al. (2016) [69]	Cylinder 100 × 200 mm	Compressive strength test Direct fire resistance test Fracture compression test
Mezzal et al. (2020) [50]	Cylinder 100 × 200 mm Cylindrical discs 150 × 65 mm Prism 100 × 100 × 500 mm	Fresh property test Compression test Splitting tensile test Ultrasonic pulse velocity Drop weight impact test Flexural toughness test Fracture energy test
Fike and Kodur (2011) [70]	Slab 152 × 152 mm × 130 mm	Fire resistance test FEM SAFIR
Bednář et al. (2013) [71]	Cube 150 × 150 × 150 mm Prism 150 mm × 150 mm × 700 mm Slab	Compressive strength test Tensile test Four-point bending test Cardington fire test
Jin et al. (2018) [51]	Cube 150 mm × 150 mm × 150 mm Beam 200 mm × 400 mm × 2800 mm Cylinder 32 mm diameter	Compressive strength test Bending test Drop hammer impact test Fire resistance test
Algourdin et al. (2020) [72]	Cylinder 150 × 300 mm Slab 600 mm × 300 mm × 120 mm Cylinder 150 mm × 35 mm	Heating test Gas permeability test Modulus of elasticity Compression test Slow heating test
Nematzadeh et al. (2020) [73]	Cylinder 100 mm × 200 mm	Compressive strength test Response surface method (RSM) Artificial neural network (ANN) approach

**Table 3:** The majority of studies examining the impact resistance properties of steel fibers.

Authors and Year	Type and Size (mm) of Specimens	Experiments Conducted
Hyun-Oh Shin et al. (2018) [80]	Cylinder 100 mm × 200 mm Prism 100 mm × 100 mm × 400 mm Railway PSC sleepers: cross-section at rail seat 265 mm × 195 mm, at center 220 mm × 180 mm; total length 2400 mm	Compression test Four-point flexure test Drop hammer impact test
Vijaya Bhaskar Reddy and Srinivasa Rao (2020) [81]	Cube 150 mm × 150 mm ×150 mm Cylinder 150 mm × 300 mm Prism 100 mm × 100 mm × 500 mm Cylindrical disc 150 mm × 63.5 mm	Compressive strength test Split tensile test Three-point flexural test Impact strength test
Nandhu Prasad and Murali (2021) [82]	Cylindrical disc 152 mm × 64 mm	Compressive strength test Falling weight impact test
Mahakavi and Chithra et al. (2019) [83]	Cube 100 mm × 100 mm × 100 mm Prism 100 mm × 100 mm × 500 mm Cylindrical disc 150 mm × 65 mm	Fresh property test Compression test Four-point bending test Drop weight impact test SEM
Azizul Islam et al. (2017) [84]	Cube 100 mm × 100 mm × 100 mm Cylinder 150 mm × 300 mm Cylinder 100 mm × 200 mm Prism 100 mm × 100 mm × 500 mm Panel 600 mm × 600 mm × 50 mm	Compressive strength Modulus of elasticity Splitting tensile strength Flexural strength Drop hammer impact test

## Data Availability

Not applicable.
